# Recent developments in filtration media and respirator technology in response to COVID-19

**DOI:** 10.1557/s43577-021-00173-6

**Published:** 2021-09-15

**Authors:** Peter L. Wang, Alex Roschli, M. Parans Paranthaman, Merlin Theodore, Corson L. Cramer, Chris Zangmeister, Yuepeng Zhang, Jeffrey J. Urban, Lonnie Love

**Affiliations:** 1 Oak Ridge National Laboratory, Oak Ridge, TN USA; 2 National Institute of Standards and Technology, Gaithersburg, USA; 3 Argonne National Laboratory, Lemont, USA; 4 Lawrence Berkeley National Laboratory, Berkeley, USA

**Keywords:** N95 respirators and filter, Manufacturing methods, Filtration efficiency, Antiviral properties

## Abstract

**Abstract:**

The COVID-19 pandemic triggered a surge in demand for N95 or equivalent respirators that the global supply chain was unable to satisfy. This shortage in critical equipment has inspired research that addresses the immediate problems and has accelerated the development of the next-generation filtration media and respirators. This article provides a brief review of the most recent work with regard to face respirators and filtration media. We discuss filtration efficiency of the widely utilized cloth masks. Next, the sterilization of and reuse of existing N95 respirators to extend the existing stockpile is discussed. To expand near-term supplies, optimization of current manufacturing methods, such as melt-blown processes and electrospinning, has been explored. Future manufacturing methods have been investigated to address long-term supply shortages. Novel materials with antiviral and sterilizable properties with the ability for multiple reuses have been developed and will contribute to the development of the next generation of longer lasting multi-use N95 respirators. Finally, additively manufactured respirators are reviewed, which enable a rapidly deployable source of reusable respirators that can use any filtration fabric.

**Graphic abstract:**

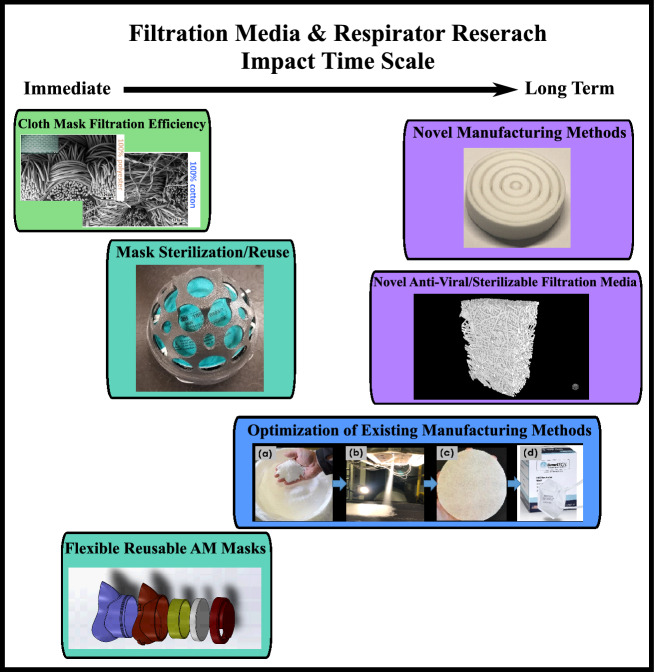

## Introduction

The severity of the COVID-19 pandemic has presented the world with an unprecedented challenge, revealing systemic weaknesses within the health care infrastructure in every country. There has been a universal shortage of personal protective equipment for health care workers,^1^ especially N95 respirators, which are vital to reducing the risk of transmission of the virus in health care settings.^2,3^ The shortage of N95 respirators has led hospitals and health care workers to reuse of and attempts to conserve old N95 respirators.^4^ Outside of medical settings, the World Health Organization (WHO) and the United States Centers for Disease Control and Prevention (US CDC) recommend to use cloth masks to reduce community spread of the virus.^5,6^

**Figure 1:**
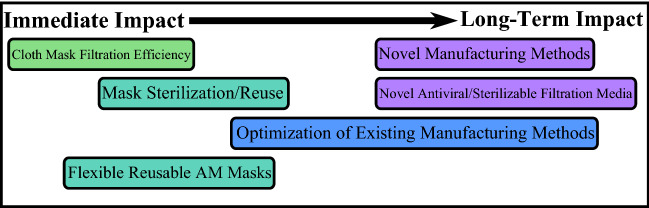
The time scale of the impact of the recent research into filtration media and respirators shows that the research performed in response to the COVID-19 pandemic covers both the immediate needs and the long-term technological advancements needed to mitigate the challenges observed in the future.

This article will review the range of research that has been performed with respect to filtration media, respirator, and respirator development in response to the COVID-19 pandemic. The research performed covers near-, medium- and long-term impacts (**Figure ****1**). For example, immediate utility can be found in cloth masks and respirator reuse studies, respirator sterilization and optimized manufacturing methods can be implemented in the time scale of months. The research into novel antiviral materials and novel manufacturing methods will have high impact on respirator efficiency and availability, but will take years to commercially deploy. The efficacy of various cloth fabrics and different implementation strategies are reviewed in this article. To extend the existing N95 inventories, technologies have been developed to enable N95 respirator reuse including sterilization and replenishing surface charge. Novel manufacturing methods and materials for N95 filter fabric have been studied and, in some cases, have led to new manufacturing lines increasing the overall supply of N95 filter fabric. Additionally, novel filter media that can be easily sterilized or has natural antiviral properties have been developed to mitigate future shortages. Finally, this article will briefly discuss the developments made in developing rapidly deployable additively manufactured respirators that could use multiple different filtration media including the newly developed filtration media.

## Testing of common materials for filter media

**Figure 2:**
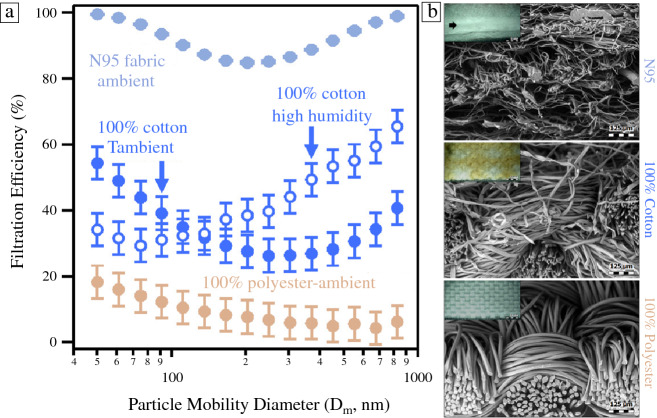
(a) Filtration efficiency versus particle size (D_m_) for each measured sample. (b) Scanning electron microscope images of each sample. Insets are multifocus visible light images by E. Vicenzi, Smithsonian Institution. Uncertainties represent 1σ.^7,8^

Zangmeister tested the filtration efficiency (FE) of more than 40 different fabric materials available for mask fabrication. These measurements were correlated with material structure using microscopy-based analysis. The FE of a fabric is a function of its fiber diameter, porosity, and thickness. Materials with small (2–5 µm) fibers have higher FE than fabrics with large (> 10 µm) fibers. FE measurements were made using mobility (size)-selected particles with diameters between 50 and 825 nm. These data showed a dramatic range in FE between materials and particle size. The measured FEs as a function of particle size of three tested materials are shown in **Figure ****2**. A single layer of melt-blown polypropylene filter material used in N95 respirators is comprised of < 5-µm fibers with high porosity and had the highest measured FE with the characteristic U-shaped FE curve predicted by filtration theory. Synthetic fabrics are typically constructed from > 20-µm fibers, which resulted in seven of the 10 tested fabrics with the lowest measured FE. Natural fabrics, such as cotton, generally exhibited the highest FE of the measured fabrics, a product of their fiber diameter (> 10 µm), thickness (0.5 mm per layer), and porosity (75%). The chaotic arrangement of fibers in natural fabrics likely also increases particle capture when compared to thinner well-ordered synthetic fabrics; see middle and bottom SEM images for comparison.^7^

The FE was tested under an environment that mimics the high humidity (90% relative humidity at 22°C) conditions a fabric experiences when used as a face covering. Under these conditions, hydrophilic materials absorbed about 10% of their mass in H_2_O. This resulted in a change in the measured FE curve when compared to the dry material, most likely due to H_2_O uptake and growth by particles (i.e., filtrate) as they traversed the material. We estimate a dry 75-nm particle grows to 300 nm, altering the shape of the FE curve. These measurements indicate that H_2_O supplied by breathing through the filter can greatly increase the FE of hydrophilic materials principally through particle growth to a size regime with higher FE.^8^

## Reuse of existing N95 respirators

The N95 standard is defined by the National Institute for Occupational Safety and Health (NIOSH)^9^ as a respirator with a filtration efficiency of 95% or greater for non-oil-containing particles. This is tested using sodium chloride with mean diameter of 0.075 ± 0.020 µm. The specification states that at a flow rate of 85 ± 2 l per minute the maximum pressure across the respirators must be 25-mm H_2_O on exhalation and 35-mm H_2_O on inhalation. This standard is similar to other standards from around the world, such as from China (KN95), Australia/New Zealand (P2), Japan (DS), and the EU (FFP2).^10^

Prior to the pandemic, N95 respirators were in great supply at low costs, so sterilization and reuse weren’t economical. Also reuse was not recommended by manufacturers as the N95 respirators are traditionally designed as one time use items. However, the onset of the pandemic caused a significant shortage in respirators causing many researchers to turn to sanitizing and other means of sterilization to lessen the burden of the respirator shortage. Additionally, the fit of the respirator is critical to its functionality and degrades with use,^11^ so the effect of the sanitation method on the fit must also be understood. Investigation into sterilization methods include UV light,^12^ steam,^13^ humid heat,^14^ autoclaves,^15^ and hydrogen peroxide.^16,17^ UV light has been used to scale production to 12,000 sanitized respirators per day.^18^ Yale researchers found that hydrogen peroxide vapor could be used for five cycles on respirators without any deformation.^19^ Researchers at the University of Manitoba tested seven different methods of sterilization, including autoclave, ethylene oxide treatment (ETO), low-temperature hydrogen peroxide gas plasma (LT-HPGP), vaporous hydrogen peroxide (VHP), peracetic acid dry fogging (PAF), ultraviolet C irradiation (UVCI), and moist heat (MH) with all methods, except ultraviolet, completely disinfecting from the virus. All respirators passed fit testing, which tests structural integrity and deformations, and filtration efficiency testing after single cycle of the sterilization methods, except LT-HPGP, which resulted in 50% failures of the respirators in filtration efficiency. VHP, PAF UVCI, and MH showed passing results in both tests after 10 cycles, while ETO passed three cycles. Sterilization using autoclave resulted in pleated layered nonwoven fabric N95 respirators that passed 10 cycles, but molded respirators failed the fit test after one cycle and the filtration efficiency testing after five cycles.^20^ Fisher et al. at NIAID studied four decontamination methods: UV light, 70°C dry heat, 70% ethanol, and VHP. VHP and ethanol showed rapid viral inactivation on N95 materials, while UV light and heat were slower and showed similar inactivation rates. N95 respirators subjected to VHP and UV maintained their fit ratings after three rounds of decontamination, while heat caused fit failure after one to two rounds, and ethanol failed after one round.^21^ In April 2020, the CDC and NIOSH named germicidal irradiation, VHP, and MH as the most promising methods of decontamination.^22^ In June 2020, the European Center of Disease Prevention and Control recommended using UV, ETO, VHP, and MH as an extraordinary last resort method to address respirator shortages.^23^ In April 2021, the CDC recommended that due to increased respirator supply, medical providers transition away from the use of decontamination methods.^24^

**Figure 3:**
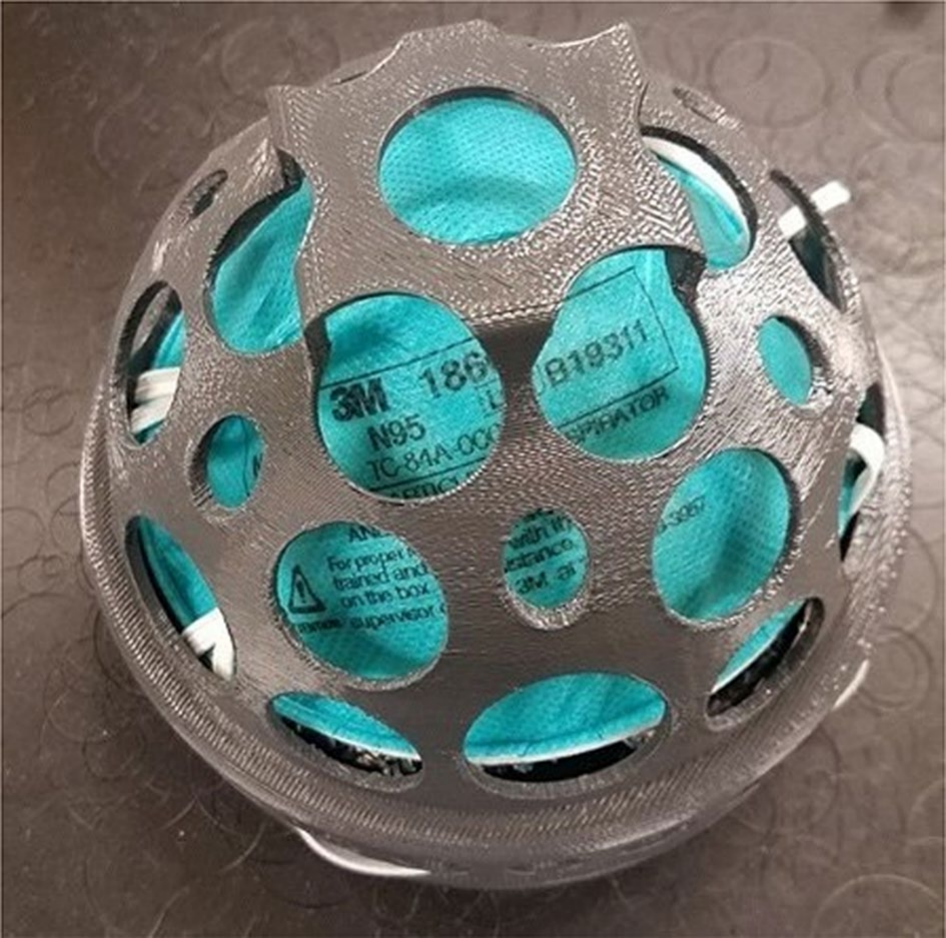
Photo of the charging station used to disinfect and apply electrostatic charge to the renewable personal protective equipment developed at Lawrence Berkeley National Laboratory. Stand allows up to 1 kV to be applied to respirators, fully renewing filtration performance for used respirators.

The primary filtration mechanism in N95 respirators relies on electrostatic trapping of virus-containing droplets within the fibrous polypropylene layers of the respirator. This allows virus trapping, despite the fact that the pore size of N95 respirators is often significantly larger than the particle size. However, this charge quickly dissipates and respirator utility declines dramatically, creating an exposure risk. The rate of dissipation can be a few hours to over a day depending on details of temperature, humidity, and use. Attempts to sterilize the respirator with heat,^25^ ionizing radiation,^26^ or disinfectant such as ethanol^27^ degrade the filtration efficiency and the structural integrity of the respirators. While UV disinfection^28^ does not degrade FE, it cannot penetrate to sterilize inner respirator layers.^26,29^ Urban and colleagues used a rechargeable test stand incorporated on a respirator cartridge (the cartridge is a removable insert within the mask, which contains the filtration media), which allows the respirator fabrics to be sterilized and recharged daily, renewing 99% of filtration efficiency. This rechargeable filter is incorporated within a custom-fit respirator that can be printed using additive manufacturing (**Figure ****3**). This enables reliable reuse of respirators; reuse also depends on mask fit, and we are collaborating with a mask fit team at Sandia National Laboratories on fit verification and optimization. The next-generation design incorporates nanoscale conductive metal fiber mixtures, which maintain an intrinsic continuous voltage, providing a self-cleansing effect that requires limited recharging. Preliminary results have shown a 10% increase in filtration efficiency combined with the ability for recharging and reuse; similar results from recharging have been found by Pirker.^26^

## Manufacturing methods and materials for filter media

The typical N95 respirator is made from two or three plies of melt-blown polypropylene (PP) electret (a dielectric material with quasi-permanent electrical charge or polarization) between two layers of spun-bond polypropylene. A corona charge is applied to the melt-blown fibers, which significantly improves filtration efficiency. While several polymers can be melt-blown into nonwoven fabrics, PP is the most widely used material due to its ease of processing, good mechanical properties, and effectiveness as a filtration device.^30,31^ The basic operation of N95 respirators or respirator is to enhance size-selective filtration capability by electrostatically charging melt-blown nonwoven PP fabrics. The electrostatic charge on the PP filter attracts and captures viral particles enabling larger porosity in respirators for easier breathing. Also, PP is a low-density polymer, hydrophobic in nature, low cost and has good chemical stability, lower melt temperatures for easy bonding, and good mechanical strength. The spun-bond PP layers (made by a process similar to melt blowing) provide the much-needed mechanical strength, while the melt-blown PP layer provides all the virus filtration features (and pressure drop for breathing) needed. The weight of the melt-blown fabric (adjusted by the thickness of the layers) and the number of plies can be varied depending on the application; however, N95 respirators typically have two plies of melt-blown PP fabrics. The application of corona charging to the melt-blown PP fabrics vastly improves the COVID virus filtration efficiency due to the formation of electrets.

## Melt-blown fabrication

**Figure 4:**
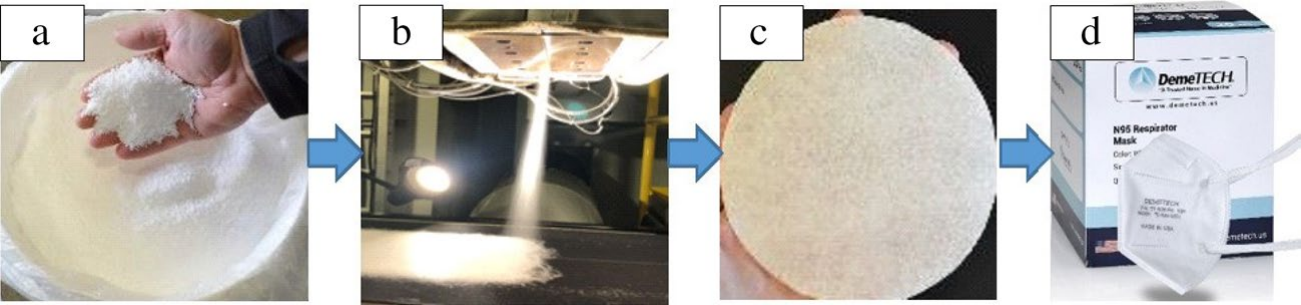
(a) Image of the starting polypropylene (PP) beads with additives.^30^ Adapted from supplemental data in; (b) Formation of PP fabrics by melt blow process through a die at Oak Ridge National Laboratory; (c) PP filters after charging; and (d) N95 respirator made out of PP filters.

Theodore, Paranthaman, and colleagues successfully identified commercial grades of PP polymer materials and demonstrated the development of N95 filtration media with the intent that the industry partners can learn quickly and help address N95 shortages.^30^ Polypropylene fabrics were manufactured by melt blowing commercial grades of polypropylene mixed with a blend of additives as shown in **Figure ****4**. The resulting fabrics were corona charged by a custom system to generate electrets and demonstrated vastly improved filtration efficiencies comparable to N95 standards. Melt-blown PP fabrics were studied in detail using advanced materials characterization techniques including differential scanning calorimetry, x-ray diffraction, scanning electron microscopy, and neutron scattering. From the analysis, we determined that the most important factor is related to crystallization of the PP polymer and the resulting electret formation. PP materials have high crystallization temperatures, larger fraction of crystallites form an efficient electret with slower crystallization rates and are effective at N95 standard filtration. Based on the FE, commercial PP materials were identified and scaled up to prepilot scale. The pressure drop observed from melt-blown PP sample was comparable to other high-performing single layer materials, which is critical for ease of breathability when used in respirators. The last but critical piece of the supply chain was converting the N95 filter media material to N95 respirators that passes NIOSH requirements for N95 respirators. The melt-blown process conditions, including the corona charging device, were transferred to the industry partner, Cummins Filtration. Cummins Filtration then supplied filter material to a few customers, including DemeTECH. The typical DemeTECH N95 respirator is comprised of five layers, with two PP filtration layers (as shown in Figure 4c) that can filter > 99% of all airborne COVID particles. The DemeTECH N95 product obtained the NIOSH approval as meeting the N95 standard and is now available to consumers and is listed on the Centers for Disease Control and Prevention of NIOSH-Approved Particulate Filtering Facepiece Respirators Manufacturers list.^32,33^ In addition, the surgical mask product, DemeMASK, is FDA approved.^34^ Based on the success with the manufacturing of PP-based N95 respirators, the team is focusing on developing the antiviral coatings. The main use of the antiviral coatings is to kill the COVID virus that are filtered on the respirator. The challenge lies on the toxicity of the material coatings and also the recyclability of the N95 respirators.

## Electrospinning fabrication

An alternative to the traditional melt-blown manufacturing method for the filtration fabric is electrospinning. This process generates ultrafine fibers ranging from less than 100 nm to a few micrometers.^35^ The diameter of these fibers can be controlled by adjusting parameters such as the polymer solutions, electric field, and distance of nozzle to collector.^36^ The fibers can then be collected into nonwoven mats with aligned, random, or mesh structures. These mats can then be used as filters in respirators.^37^ Numerous synthetic polymers such as poly(vinyl alcohol) (PVA), poly(ethylene oxide) (PEO), bioactive glass, poly(vinyl pyrrolidone) (PVP), poly(lactic acid) (PLA), and poly(ε-caprolactone) (PCL) and natural biopolymers such as thermoplastic carboxymethyl cellulose, chitin, chitosan, starch, pullulan, cinnamon oil, and whey protein have been used in the electrospinning process to manufacture filtration materials with some showing bioactive properties.^10^ Zhang et al. demonstrated a spider-web-inspired electrospun filter with filtration efficiency for 300-nm particles of 99.995% and pressure drop of less than 88.5 Pa.^38^ Using six layers of filter fabric made from charged poly(vinylidene fluoride) (PVDF) nanofibers (525 nm), a filtration efficiency of 92% for 50 nm particles and 98% for 300 nm particles was demonstrated with a pressure drop of 26 Pa.^39^ Poly(ethylene terephthalate) (PET) recovered from domestic waste streams was used in an electrospinning process to produce a fabric with a filtration efficiency of 98% for 120-nm particles.^40^ Efforts to reduce the environmental impact of mass respirator and filter consumption have led to developments in biodegradable filtration media made from cellulose acetate and cationic surfactant cetylpyridinium bromide.^41^

## Novel filtration media

Novel filtration media have been developed that has intrinsic properties that prolong the use time of the respirators such as antiviral properties or fully washable and serializable with minimal degradation. These materials can improve the overall safety of the respirators as well as reduce the biowaste produced through reduction of respirators consumed.^42^

## Antiviral filtration media

The antiviral components can be metal-based nanoparticles such as silver, gold, and oxide-based CuO, ZnO, TiO_2_, and SiO_2_ nanoparticles; nonmetals such as carbon dots, carbon nanotubes, 2D graphene oxide, and NaCl; and organic compounds such as Nhalamine, Chitosans, and photodynamic materials such as zinc-tetra(4-*N*-methylpyridyl)porphine, which produces reactive oxygen species to kill pathogens.^43^ Recently, these materials have been incorporated into polymer filtration media to give N95 respirators antiviral properties.^44,45^ Copper sulfide (CuS) has been impregnated in nylon at 4.4% and 17.6% (w/w) in the outer and inner layers, respectively, in a three-layer respirator. The respirator showed nearly complete virus deactivation after 1 h as observed by cytopathy, fluorescence, and viral copy number.^46^ A shellac and copper nanoparticle spray coating applied to nonwoven PP surgical respirators was shown to increase hydrophobicity of the respirators and 2–3 log decrease of viral concentrations after 5 min of solar exposure. The respirators maintained their filtration efficiency after five treatment cycles.^47^

Hydrophobic polyacrylonitrile (PAN) and hydrophilic poly(vinyl alcohol-*co*-ethylene) (PVA-*co*-PE) were blended with vitamin K compounds (VK) and electrospun into a nonwoven nanofibrous fabric, which generated reactive oxygen species (ROS) under daylight and ultraviolet. After less than 90 min of daylight exposure, the 99.9% of the viruses had been eliminated. The PVA-*co*-PE generated the ROS at a higher rate than the PAN and maintained its antiviral functions after five repeated exposures to viruses and light irradiation cycles, suggesting that it is better suited as an antiviral respirator material.^48^ Cotton respirators were given antiviral properties by first modifying the cotton with 2-die-thylaminoethyl chloride (DEAE-Cl) then functionalized with Rose Bengal (RB) or anthraquinone-2-sulfonic acid sodium salt mono-hydrate (2-AQS). The RB and 2-AQS were added through a traditional dying process. The functionalized cotton produced ROS under daylight, which showed increasing effectiveness with higher concentrations of RB or 2-AQS, with 50 mg/L RB or 250 mg/L 2-AQS after 30 min of daylight exposure 6 log reduction of viral equivalent were observed, and with 500 mg/L of 2-AQS the same 6 log reduction was observed after 10 min of daylight exposure or 60 min in the dark. The RB and 2-AQS maintained their efficacy after seven days of continuous light exposure, while only the RB maintained its antiviral properties after standard hand washing cycles.^49^

**Figure 5:**
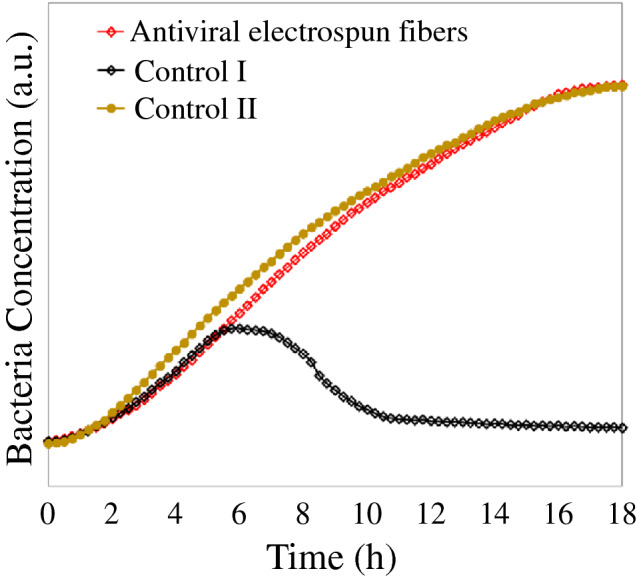
Antiviral test of the antiviral electrospun fibers and two control samples, where Control I is CuAg-free electrospun fibers and Control II is phage-free bacteria solution.

Zhang and colleagues developed nanofiber face respirator filters with 95% filtration efficiency using electrospinning technology. These fibers contain antiviral ingredients such as CuAg and showed virus inactivation effect after a short exposure of 15 min. The antiviral tests were based on Phi6 phage, a SARS-CoV-2 virus surrogate. During tests, CuAg-containing electrospun fibers were examined together with two control samples shown in **Figure ****5**. Control I were CuAg-free fibers. This cohort was exposed to Phi6 phage for 15 min with the CuAg-containing fiber samples before being transferred to a bacteria culture. The antiviral tests showed a reduced bacteria population for Control I after six hours of bacteria culturing, indicating a phage attack on the bacteria in this sample (i.e., Control I did not affect phage). Control II was a bare bacterial solution that did not contain any fibers. This sample was not exposed to Phi6 phage and showed bacteria duplication over time. The fact that the CuAg-containing fibers showed a similar behavior to Control II, but were different from Control I suggests that the phage was effectively inactivated by the fibers.

## Ceramic filter media

Typically, air filtering media for masks, respirators, and even vehicle air supplies are made from polypropylene fibrous membranes because it is effective at trapping small particles and aerosols. However, these materials rely on electrostatic charges for most effective use, and the charge degrades as the filters are used. One alternative for filter media is porous ceramic which do not rely on charges to filter through powder processing techniques. The ceramic filters can withstand repeated cycles of autoclave sterilization without significant degradation allowing for repeated reuse far beyond any polymer fiber filters. Ceramics made with particulate processing provide a microstructure capable of filtering air, and this can be controlled based on microstructure.^50–52^ Ceramics have also been used to filter out viruses and bacteria in water.^53,54^ The biggest use of ceramics in filtration has been with diesel particulates, though, where small particles on the nano and submicron scale were filtered out with ceramics such as SiC, Al_2_O_3_, and Cordierite.^55,56^ One study shows that the pore sizes (4–5 µm) can be smaller than this study’s pore sizes (25–100 µm) and still achieve the desired pressure drops.^57^ Another study found that pores of 20–50 µm would work well for diesel particulate filters with more open macroporosity and channels.^58^

The design of ceramic filter media is enhanced with additive manufacturing (AM) through increased surface area and inner channel networks affecting pressure drops.^59,60^ The ability to make the filters with AM allows for the use of complex geometries that can increase the overall filter area to lower pressure drop and allow for breathability in masks and respirators while maintaining a small overall footprint when making the material. Cramer and colleagues made ceramic filters media from alumina powder and binder jet 3D printing (BJ3DP). Because of the large particles needed for BJ3DP, complex filters were printed and post-treated with a colloidal silica infiltration and subsequent sintering to achieve fully consolidated, but porous, alumina silica and alumina mullite composite parts. The complex filters that had increased surface area provided an acceptable pressure drop for N95 masks.

**Figure 6:**
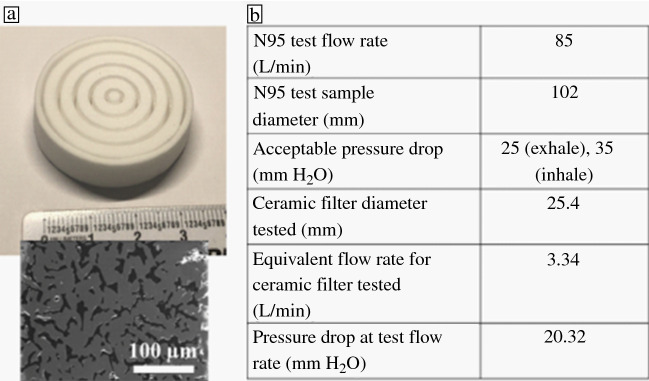
Research done at Oak Ridge National Laboratory on 3D-printed ceramic filters where (a) is the macron and microstructure of an alumina silica filter and (b) is the pressure drop data comparing the pressure drop to N95 tests where the equivalent pressure drop for the smaller 3D-printed sample passes the criteria for N95.

The N95 NIOSH standard test for pressure drop is performed on a sample 102 mm in diameter.^61^ For the tests, the flow rates were scaled based on the printed diameters to ensure a constant air velocity, so 3.34 L/min of airflow was used. The complex filter made in BJ3DP had a pressure drop of 20.32 mm H_2_O and thus would be acceptable as N95 material. **Figure ****6** shows the complex, printed filter and microstructures as well as a table for the pressure drop analysis for the filters. The 3D-printed filters pass the test for N95 pressure drops; thus, they could be used in masks. Needless to say, these filters can be used in respirators, masks, ventilators, and even vehicles, but more testing of the virus and particulate trapping must be done.

## Reusable respirator technologies

The limited supply of respirators and filtration media has inspired the development of novel reusable additively manufactured respirators that are filter media agnostic because of the widespread availability of desktop 3D printers. A respirator adapter was developed that allowed N95 respirators to be cut into quarters and while still adhering to the N95 standard.^62^ This simple adapter alone was sufficient to quadruple the supply of respirators. Many open-source face respirator designs were found online that they could print and test with a variety of commonly available filters.^63^ Three-dimensional printing and CT scanning was used to develop respirator designs with the ideal face seal and fit.^64^ Makers across the globe have worked to create reusable 3D-printed face shields with commodity materials.^65^ These face shields helped to conserve the N95 respirator supply by protecting respirators from sneezes and spills that would contaminate the body of the respirator.

**Figure 7:**
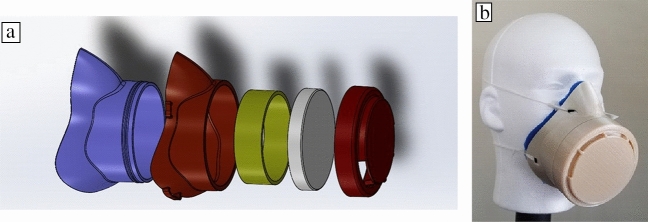
(a) Assembly of all components needed to make the Oak Ridge National Laboratory prototype mask. Red and yellow components are rigid 3D-printed, white is the filter sheet, and blue is a soft silicone inner liner. A flexible liner is shown in blue. (b) Fully assembled mask prototype where the filter is sandwiched between the end cap and main body of the mask.

Roschli and colleagues performed prototyping work to demonstrate how masks could be made to use commodity filter material, such as that of a home HVAC filter. This was done by 3D printing a mask assembly that conformed to the face and sandwiched a sheet of material between the mask body and cap. **Figure ****7** shows all of the components needed to assemble this prototype mask and the fully assembled mask.

## Conclusion

The COVID-19 Pandemic has exposed the global supply chain’s inability to address a significant surge in demand. The studies performed on existing respirator technologies, including both cloth and N95 respirators will provide critical data that will inform future pandemic policy. The research into sterilization and reuse of respirators as well as additively manufactured respirators have enabled rapid deployment of these technologies to help alleviate supply chain shortages. However, the long-term solution requires the development of novel respirator materials and material manufacturing methods. The research and development of filter media with antiviral properties and increased use life, as well as the deeper exploration into the melt-blown, electrospinning, and AM ceramic processing has revealed numerous paths forward that would provide a significant improvement over the current commercially available options. These advancements will help usher in the next generation of personal protective equipment, which will prove invaluable for medical practitioners and the general population in the future.
